# A statistical geometry analysis of simulated water-DMSO and water-MeCN binary mixtures for biomolecular studies

**DOI:** 10.6026/97320630014350

**Published:** 2018-07-31

**Authors:** Neetu Singh Yadav, Devapriya Choudhury

**Affiliations:** 1 School of Biotechnology, Jawaharlal Nehru University, New Delhi-110067, India;

**Keywords:** Statistical geometry, Microheterogeneity, Molecular Dynamics

## Abstract

Water-Dimethylsulfoxide (DMSO) and water-Acetonitrile (MeCN) binary mixtures at various molar ratios ranging from 0 to 1 are studied using Molecular Dynamics (MD) simulations. Hydration properties of water in different regions of MeCN/DMSO are investigated by using the statistical geometry approach. The obtained results reveal that in water-DMSO simulations both water and solvent molecules prefer to be in mixed cluster forms, depending upon the concentration of DMSO. While in case of water-MeCN mixtures, self-association of water and acetonitrile molecules, take place, showing microheterogeneity associated with the water- MeCN binary mixtures. The results highlight the utility of statistical geometric analysis of MD simulation data of binary liquid mixtures for rapid screening of polar organic solvents in non-aqueous enzymology.

## Background

A key element of research in non-aqueous enzymology is therefore to screen for suitable organic solvents that have minimal effects on the structure of the enzyme of interest, or to screen for enzymes that are stable when high concentrations of polar organic solvents (POS) are added to the surrounding medium. While there have been a number of spectacular successes of the experimental screening approach [1], it is also important to develop in silico screening methods for POSs suitable for a particular protein, or even a rational approach to suggest mutations that can enable an enzyme protein to resist the harmful effects of added POSs. Molecular Dynamics (MD) simulations provide a promising approach for the study of enzymes in non-aqueous media. The force-field parameter set in use with much popular MD software has recently been enhanced with parameters for a large number of organic solvents [2, 3]. However, the very large number of particles that need to be taken into account in realistic MD simulations of proteins dissolved in partially for fully non-aqueous systems require immense computational resources and often an inordinate amount of time, making a large scale MD based solvent screening program nonfeasible.

A fairly large number of studies have accumulated that convincingly demonstrate that the deleterious effects of POSs on proteins stems from the effects that these solvents exert on the large-scale structure of water [4]. Hence one way to speed up the simulations would be to only consider mixtures of water and POSs and monitor the time-averaged large-scale structure of the mixtures and compare them with that of liquid water. Simply using mixtures of water and POSs would greatly reduce the particle numbers in the simulation and thus cut down the time required for analysis by a significant amount. We test this hypothesis by analyzing MD simulations of binary mixtures water and two organic solvents viz., Acetonitrile (MeCN) and Dimethyl sulphoxide (DMSO), both of which are known to significantly affect the structure of proteins dissolved in them. We also tested the feasibility of using methods of statistical geometry to analyze the large-scale time-averaged structures of the binary mixtures as obtained from standard MD simulations.

Application of statistical geometry for the analysis of the large scale structure of molecular liquids were first developed by Bernal and others in the 1950s and was found to be a promising tool for the analysis and prediction of a number of experimentally measurable quantities [5]. Unfortunately, except for a few notable exceptions [6, 7], these methods have not been routinely used in bio-molecular simulations.

## Systems and Methods

### Molecular Dynamics Simulations

A series of MD simulations were carried out for mixtures of water and POS (MeCN and DMSO) where the concentration of the organic solvent was varied. Details of the MD simulation protocol and its validation are given in the supplementary data.

### Statistical Geometry

**Figure 1:**
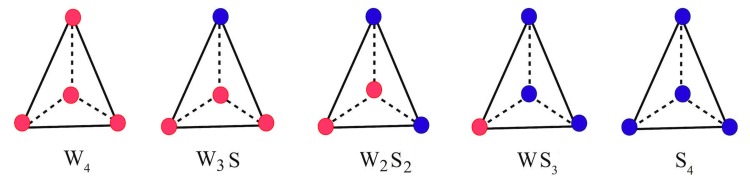
Five types of water-co-solvent association patterns expressed as vertices of Delaunay tetrahedral. Red and Blue colors denote water (W) and POS (S) atoms respectively.

Statistical Geometry analyses were initiated by first carrying partitioning the space contained in the simulation box by means of Delaunay Tessellation [5]. Delaunay tessellation in 3D partitions space defined by a cloud of points into a series of nonoverlapping tetrahedral. The vertices of the tetrahedral are defined using the cloud of points given as input. Points that are not a vertex of a particular tetrahedron are not allowed to be anywhere within the volume in the tetrahedron. In our case, every non-hydrogen atom contained within the simulation box defined the clouds of points. Since the tetrahedral obtained by Delaunay tessellation do not contain any other point within them, points across any edge of a given tetrahedron are by definition nearest neighbors. We used this property to carry out nearest neighbor analysis of different atoms in our binary mixtures. For this we considered only two types of atoms, the first type denoted by W consisted of only the Oxygen atoms of water molecules and the second type, denoted by S could consist of any non-hydrogen atom from the POS, which in case of MeCN could either be carbon or nitrogen, or carbon, oxygen and sulphur in case of DMSO. With these designations, we can have five types of tetrahedral depending on the identity of the vertices, there are: W4, i.e., composed of four W atoms, W3S, composed of three W and one S atom and similarly, W2S2, WS3 and S4 Figure 1. We then counted the numbers of each class of these tetrahedral from every snapshot of our MD simulations.

The numbers were further normalized in terms of a log-odds ratio (f) as follows:

f_i_ = P_i,obs_ / P_i,exp_

*P_i,obs_* denotes the observed probability of finding a particular class of tetrahedron and is given by:

P_i,obs_ = n_i_ / ∑ n_i_

where, *n_i_* denotes the number of tetrahedral belonging to the ith class.

*P_i,exp_* denotes the expected probability of finding a particular class of tetrahedron and is given by:

P_i,exp_ = 4_k_p_w_ (1-pw)^(4-k)^

where, k (0 ≤ k ≤ 4) denotes the number of w atoms in the particular tetrahedron class, and p_w_ denotes the probability of finding a w atom and is given by:

P_w_ = n_w_ / n_T_

where, n_w_ denotes the number of water oxygen atoms and n_T_ denotes the total number of non-hydrogen atoms in the simulation box.

The local structure of the mixture can be quantified by the distortion of the Delaunay tetrahedral, this has been defined in terms of a parameter called tetrahedrality, which has been defined as follows [8]:

T = ∑ ((l_i_ - l_j_)^2^ / 15 l̄^2^)

A perfectly regular tetrahedron has a tetrahedrality of 0, which increasing deviations from regularity causes a corresponding increase in the parameter.

## Results and Discussion

**Figure 2:**
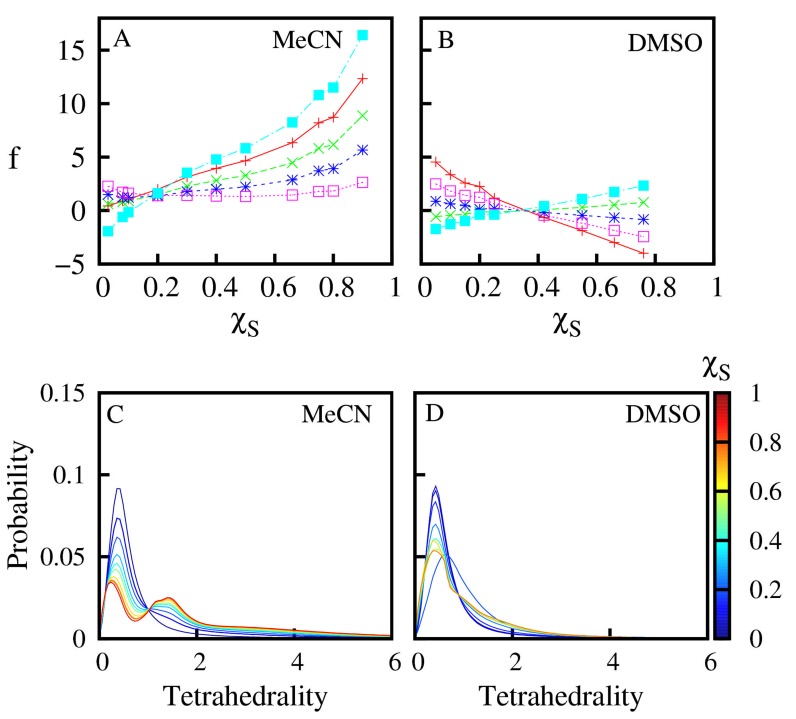
Over (under) abundance of different water-solvent association patterns in water-MeCN (A) and water-DMSO (B) mixtures as a function of MeCN and DMSO concentration (χS). A positive Log-Odds value (f) indicates a greater than expected occurrence of a particular association pattern and vice-versa for negative f values. Red, Green, Blue, Pink and Cyan color lines denotes W4, W3S, W2S2, WS3 and S4 types of tetrahedron respectively. Panel C, D plots the distribution of Tetrahedrality values for all water-solvent tetrahedral with increasing concentration (χS) of MeCN (Panel C) and DMSO (Panel C). For this, all types of tetrahedral were clubbed together and their tetrahedrality values were calculated.

Figure 2A and 2B and plots the log-odds ratio (f), for different tetrahedron types as function of added solvent concentration. As can be seen from Figure 2A, in case of MeCN, the log-odds ratio for W4 and S4 types of tetrahedral become increasingly dominant with increasing MeCN concentration. This indicates that waterwater and MeCN-MeCN associations are more preferred over water-MeCN associations. In case of DMSO, the picture is quite different, firstly none of the tetrahedron classes dominate to the extent as W4 and S4 types dominate in case of MeCN. Furthermore, the W4 type shows a significant decline. The preference of water-water self-association in water-MeCN binary mixtures had been previously suggested both from experimental [9, 10, 11, 12] as well computational [13, 14] suggest that clusters of pure water and pure MeCN can co-exist in binary mixtures, creating a phenomenon termed "microheterogeneity". The dominance of W4 and S4 type tetrahedral over the W3S, W2S2 and WS3 types strongly supports these observations.

In case of DMSO-water binary mixtures, some previous studies suggested the presence of (H2O)2:DMSO type of complexes at low concentrations of DMSO (χDMSO ~0.33) and (DMSO)2:H2O clusters at high concentrations (χDMSO ~0.6) [15], while others suggested the presence of clusters with a complex composition of water and DMSO [16]. Thus it can be concluded that significant amount of water-DMSO interaction occurs leading to water-DMSO complexes of various stoichiometries. Our statistical geometry analysis supports this view by showing that none of the five types of tetrahedral dominate to the extent observed in water- MeCN mixtures. The small but significant decline of W4 tetrahedral points towards the possibility that pure water clusters are disfavored in water-DMSO binary mixtures.

The local spatial arrangement of the binary mixtures can be gauged from the tetrahedrality distributions shown in Figure 2C and 2D. In pure aqueous medium or in the presence of low concentrations of organic solvents, the peak of the tetrahedrality distributions are close to 0, indicating the dominance of nearly regular tetrahedral. This is expected behavior, since water molecule tends to make hydrogen bonded clusters with four other water molecules, and these hydrogen bonds are directed towards the vertices of a regular tetrahedron. As the organic solvent content increases, the water-water hydrogen bond network begins to get disrupted which leads to increasing distortion in shape of the tetrahedral. What is interesting however is that in case of water-DMSO binary mixtures, increase in organic solvent content merely increases the spread of the tetrahedrality values. However, the dominating component is made up of nearly regular tetrahedral. In case of water-MeCN mixtures, the distributions turn bi-modal with a second peak with a tetrahedrality value near 1.75 becomes co-dominant with the first peak with tetrahedrality value near 0. An explanation of this difference becomes apparent when the tetrahedrality distribution for each class of tetrahedral is calculated separately (Figure S2 - see supplementary data). The figure shows that in water-DMSO binary mixtures, the tetrahedrality distribution of solvent free W4 type of tetrahedral gets perturbed with increasing concentrations of DMSO. This behavior is not seen in case of water-MeCN mixtures. The perturbation in the tetrahedrality distribution of W4 tetrahedral by DMSO indicates that the solvent can interfere with water-water association network. It should be noted that the water molecules that are participating in a W4 tetrahedron could also be part of other mixed water-DMSO tetrahedral simultaneously. The perturbation of W4 tetrahedral may therefore be due to the fact that molecules involved the formation of pure-water cluster, simultaneously be part of neighboring water-DMSO clusters, causing a spatial perturbation in the water-water interaction geometry. The geometry of pure S4 tetrahedral also shows a dramatic concentration dependent change. At high water contents, there is a very sharp peak in the S4 tetrahedrality distribution indicating a very specific DMSODMSO interaction geometry. With increase in DMSO content various other forms of interactions develop with the consequent increase in spread of the tetrahedrality values. Among the mixed type of tetrahedral, i.e., W3S, W2S2 and WS3, in water-DMSO mixtures, one finds increase in DMSO content progressively increase the spread of tetrahedrality values. Taken together, one observes a diversity of water-DMSO association patterns in binary mixtures, which result in the observed spread of tetrahedrality values.

In case of MeCN the situation is quite different, there is almost no change in the shape of the tetrahedrality distribution of W4 and W3S types of tetrahedral. While W2S2 and WS3 types show a clear bi-modal distribution with increasing organic solvent concentration. In case of the S4 tetrahedral, type distribution is quite spread out, but one the modes observed in W2S2 and WS3 distribution is clearly visible. The observed pattern indicates that there is a specific pattern of MeCN-MeCN association which is getting more pronounced with increasing MeCN concentration. X-ray diffraction studies [12] indicate the formation of specific zig-zag clusters of MeCN formed bi dipolar interactions both in the pure liquid as well as in binary mixtures with high solvent content. The role of tetrahedral with tetrahedrality of 1.75 in the formation of MeCN clusters is for further investigation.

## Conclusion

Statistical geometric analysis of MD simulation data of binary mixtures was able to quickly detect significant differences in the structure of water-DMSO and water-MeCN binary mixtures. One can, in principle, apply the method of MD simulations to large number of binary mixtures with different organic solvent components. Statistical geometric analysis on the simulation data would provide a common set of parameters to classify such binary mixtures into different categories, and one or a few examples from each category can be subjected to further intense study regarding their effects on protein structures. The combinatorial problem of a large number of solvents and a large number of concentration regimes for each solvent is mitigated. Thus, it will be a reasonably rapid tool for screening of organic solvents in non-aqueous enzymology.

## Supplementary material

Data 1

## Abbreviations

POS: Polar Organic Solvent
χS: Mole fraction of Solvent
