# Supplementary material for: A statistical geometry analysis of simulated water-DMSO and water-MeCN binary mixtures for biomolecular studies
Source: Bioinformation. 2018 Jul 31;14(7):350–6. doi: 10.6026/97320630014350 (PMC6143356; doi:10.6026/97320630014350)
Supplement: Data 1 [file 97320630014350S1.pdf]

BIOINFORMATION
Discovery at the interface of physical and biological sciences

OPEN ACCESS GOLD

Supplementary Data:

Methodology:
Two sets of MD simulations were performed. In first set, twelve MeCN-water mixtures were simulated. The concentration of MeCN was varied from mole-fraction of 0.03 to 1. In case of DMSO, eleven simulations were carried out where the concentration of DMSO was varied between molefractions 0.05 to 1. For comparison, a simulation of pure water was also carried out.

The number of water and POS required for a particular concentration were calculated by the following formula:
$C_{vol}= (M_{os}\rho_{os})n_{os} / (M_{os}\rho_{os})n_{os}+(M_w\rho_w)n_w$
Where, M, ρ, and n represent the molecular weight, density and number of molecules respectively.

Subscripts os and w denotes organic solvent and water respectively. In case of water-DMSO mixtures, the density of the system, below 50 % v/v DMSO is ~1 gm/ml which increases to 1.1 gm/ml at concentrations >50 % v/v DMSO. However, in water-MeCN, the density of the system decreases from 1 gm/ml with increasing MeCN concentration. Adequate numbers of water, DMSO and MeCN, at indicated concentrations were placed randomly, in a cubic box by using PACKMOL **[1]**. The number of Water, DMSO and MeCN molecules, along with the edge length of boxes are given in Tables S1, S2. The TIP4P model **[2]** of water and for the organic solvents, GAFF force-field parameters **[3]** was used. All atom parameters for DMSO, were downloaded from the website (www.virtualchemistry.org) **[4],** while for MeCN the parameters were taken from (http://research.bmh.manchester.ac.uk/bryce/amber/) site **[5].**

The simulation protocol consisted of three major steps: (a) In the first step, the box was energy minimized for 10,000 steps using steepest Descent method. (b) In the second step, the equilibration ran for 15 ns in isothermal-isobaric (NPT ensemble). During equilibration, the temperature and pressure were kept constant by using Berendsen's thermostat (τt) and barostat (τp) with time constants for temperature and pressure held at 0.1ps and 1.0ps respectively. (c) The production simulation was carried out for 15 ns under constant volume and temperature. Structures were saved at every ps. Equations of motion were integrated using the leapfrog algorithm, with time-step of 1 fs. Short-range electrostatics cut-off was set as 15Å. Particle-Mesh Ewald algorithm was used to account for long-range electrostatics **[6].** van der Waal's energy cut-off was set at 15Å with dispersion corrections on energy and pressure. Neighbour lists were updated at every 10th step.

Validation of MD simulations:
MD simulations were validated by calculating various concentration dependent physico-chemical parameters like density, viscosity, self-diffusion coefficient and compared with experimental values (**Figure S1**). In case of water-DMSO binary mixtures as the number of DMSO increases the density of system decreases (**Figure S1A**). The calculated density values are continuously over predicted from experimentally observed values with the error rate of 2.1-4.8%. While in the case of Water-MeCN binary mixtures, density of system decreases linearly with the rise in concentration of MeCN (**Figure S1B**), here the calculated density values are under-predicted as compared to experimental values but trend wise, they are similar to the experimentally observed values **[7].** The self-diffusion coefficient is a dynamic property of the system. In case of water-DMSO binary mixtures, diffusion curve of water and DMSO molecules shows a progressive decrease up-to **χ**DMSO ~0.33, but further increment of DMSO (**χ**DMSO ≥ 0.3) concentration seems to have no effect on the diffusion of both molecules (**Figure S1C**). In the water-MeCN binary mixture, the diffusion of water decreases from up-to **χ**MeCN **~**0.4. While at high MeCN concentration, the diffusion remains unaffected by MeCN molecules (**Figure S1D**). Unlike, water the diffusion of MeCN molecules, in the water-MeCN binary mixture, shows no such drastic reduction. Here, the diffusion values remain constant between 2.92 to 3.98, overall the concentration range.

The viscosity variation of water-DMSO and water-MeCN binary mixture (**Figure S1 E, F** respectively), apart from showing a linear decrease, as in experimental values **[8, 9],** is showing a zig-zag pattern with long error bars. This high error may be due to either the deficiencies of the parameters, or the improper equilibration of the system. The other possible reason for this absurd behavior of the viscosity variation may be the slow convergence of the formula, because of the fluctuation of the pressure and volume in the simulation box. Though the pressure of the system was kept constant in the NPT ensemble, still remarkable fluctuations occur throughout the simulation, which leads to the asymptotic behaviour of the pressure autocorrelation **[10].**

The calculated values of various physico-chemical parameters of the binary mixtures except perhaps for viscosity are in reasonable agreement with the experimental results, hence simulations were considered accurate enough for further analysis **(Figure S1).**

Table S1: Number of DMSO and Water molecules in the cubic simulation box.

Mole-Fraction (DMSO)	Water Numbers	DMSO Numbers	Box Vectors (nm)
0.0	1728	-	3.72, 3.72, 3.72
0.05	1298	86	3.65, 3.65, 3.65
0.1	1071	130	3.60, 3.60, 3.60
0.15	924	163	3.58, 3.58, 3.58
0.2	784	198	3.63, 3.63, 3.63
0.25	670	224	3.72, 3.72, 3.72
0.3	594	244	3.55, 3.55, 3.55
0.42	918	660	4.66, 4.66, 4.66

BIOINFORMATION
Discovery at the interface of physical and biological sciences
OPEN ACCESS GOLD

0.55	440	550	4.22, 4.22, 4.22
0.66	320	660	4.39, 4.39, 4.39
0.76	230	727	4.46, 4.46, 4.46
1.0	-	1000	4.82, 4.82, 4.82

Table S2: Number of MeCN and Water molecules in the simulation box.

Mole-Fraction (MeCN)	Water Numbers	MeCN Numbers	Box-Vector (nm)
0.0	1728	-	3.72, 3.72, 3.72
0.03	1000	40	3.50, 3.50, 3.50
0.08	900	80	4.00, 4.00, 4.00
0.1	900	100	4.00, 4.00, 4.00
0.2	800	200	4.00, 4.00, 4.00
0.3	680	340	4.00, 4.00, 4.0
0.4	570	420	4.00, 4.00, 4.00
0.5	430	430	3.50, 3.50, 3.50
0.66	280	550	4.00, 4.00, 4.00
0.75	150	550	3.82, 3.82, 3.82
0.8	120	520	4.00, 4.00, 4.00
0.9	50	550	4.00, 4.00, 4.00
1.0	-	500	3.62, 3.62, 3.62

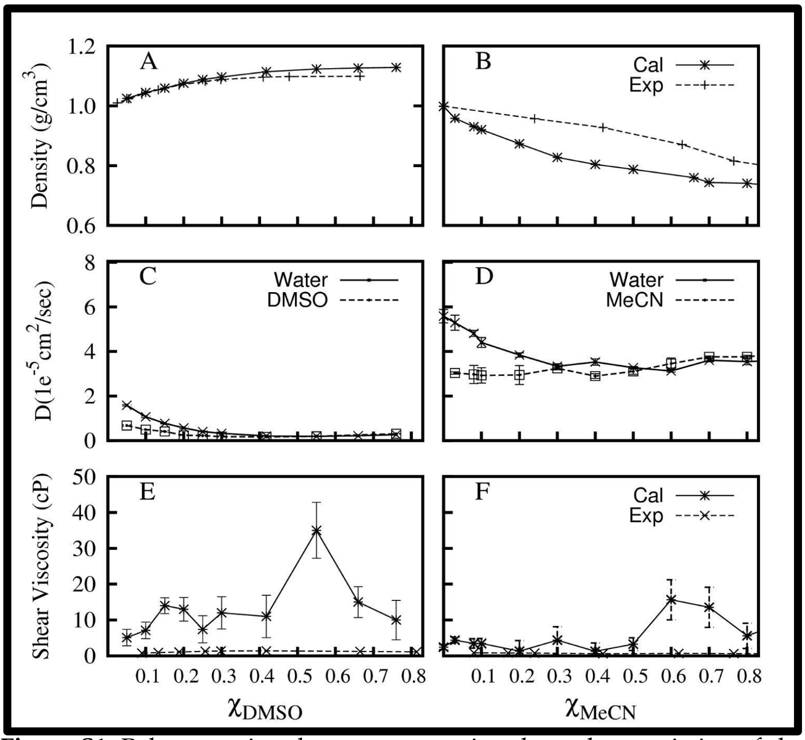

Figure S1: Polar organic solvent concentration dependent variation of densities (A: DMSO, B: MeCN), Self Diffusion Coefficient (C: DMSO, D: MeCN) and Viscosity (E: DMSO, F: MeCN). In case of water-DMSO binary mixtures the experimental density values, for density and viscosity were taken from ref **[11],** while for water-MeCN experimental values were obtained from ref **[7].**

BIOINFORMATION
Discovery at the interface of physical and biological sciences

OPEN ACCESS GOLD

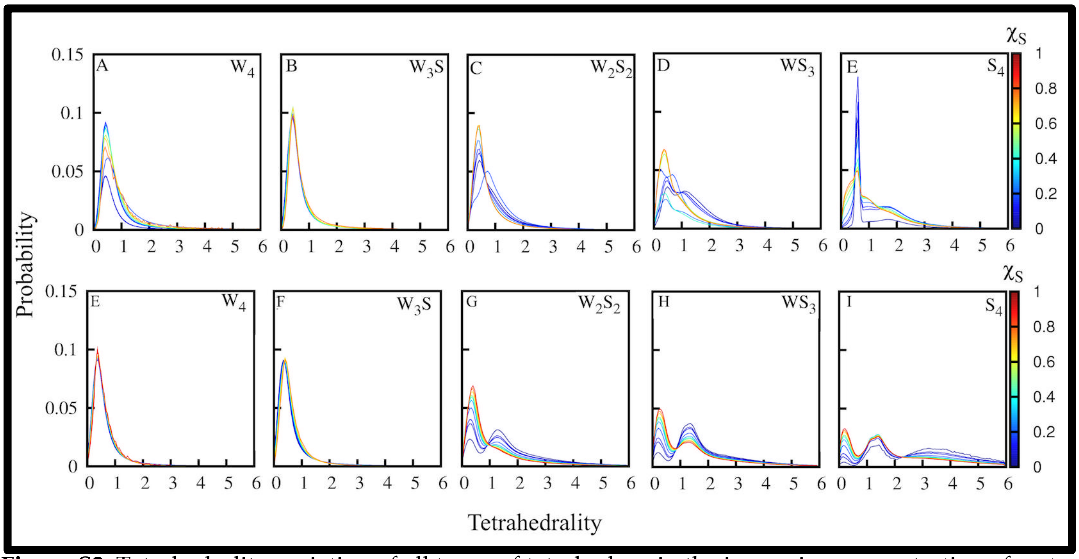

Figure S2: Tetrahedrality variation of all types of tetrahedron in the increasing concentration of water-DMSO (Panels A to E) and water-MeCN (Panels E to I). The color bar indicates the concentration (χS).

References:

[1] Martínez L *et al.* J Comput Chem. 2009, **30:**2157. [PMID: 19229944]
[2] Jorgensen WL *et al.* J Chem Phys. 1983, **79:**926.
[3] Wang J *et al.* Journal of Computational Chemistry. 2004, **25:**1157. [PMID: 15116359]
[4] Luzar A & Chandler D. J Chem Phys. 1993, **98:**8160.
[5] Grabuleda X *et al.* J Comput Chem. 2000, **21:**901.
[6] Darden T *et al.* J Chem Phys. 1993, **98:**10089.
[7] Cunningham GP *et al.* J Chem Eng Data, 1967, **12:**336.
[8] Gabrielian L & Markarian S. J Mol Liq. 2004, **112**:137.
[9] Tsierkezos NG *et al.* J Chem Eng Data. 2000, **45:**395.
[10] Hess B. J Chem Phys. 2002, **116:**209.
[11] Cowie J & Toporowski P. Can J Chem. 1961, **39:**2240.

OPEN ACCESS GOLD

BMIS
Biomedical Informatics Society

Agro Informatics Society
AGIS

Journal
